# OncoImmune machine-learning model predicts immune response and prognosis in leiomyosarcoma

**DOI:** 10.17305/bb.2025.12342

**Published:** 2025-06-04

**Authors:** Jingrong Deng, Changfa Shu, Dong Wang, Richard Nimbona, Xingping Zhao, Dabao Xu

**Affiliations:** 1 Department of Obstetrics and Gynecology, The Third Xiangya Hospital of Central South University, Changsha, Hunan, China; 2 Branch of National Clinical Research Center for Obstetrics and Gynecology, The Third Xiangya Hospital of Central South University, Changsha, Hunan, China; 3 Center for Gynecological Disease and Reproductive Health, Furong Laboratory, Changsha, Hunan, China; 4 Department of Orthopedics, The Third Xiangya Hospital of Central South University, Changsha, Hunan, China

**Keywords:** Leiomyosarcoma, LMS, monocyte differentiation, *ATRX*, immune response, machine learning, ML

## Abstract

Leiomyosarcoma (LMS) is one of the most aggressive tumors originating from smooth muscle cells, characterized by a high recurrence rate and frequent distant metastasis. Despite advancements in targeted therapies and immunotherapies, these interventions have failed to significantly improve the long-term prognosis for LMS patients. Here, we identified OncoImmune differentially expressed genes (DEGs) that influence monocyte differentiation and the progression of LMS, revealing varied immune activation states in LMS patients. Using a machine learning (ML) approach, we developed a prognostic model based on OncoImmune hub DEGs, which offers moderate accuracy in predicting risk levels among LMS patients. Mechanistically, we found that *ATRX* mutation may regulate coiled-coil domain-containing protein 69 (*CCDC69*) expression, leading to functional alterations in mast cells and immune unresponsiveness through the modulation of various immune-related signaling pathways. This ML-based prognostic model, centered on seven OncoImmune hub DEGs, along with *ATRX* gene status, represent promising biomarkers for predicting prognosis, molecular characteristics, and immune features in LMS.

## Introduction

Leiomyosarcoma (LMS) is a malignant tumor characterized by smooth muscle cell differentiation and can develop in various parts of the body, including muscle, the gastrointestinal tract, and the uterus. It is one of the most common subtypes of adult soft tissue sarcoma [1, 2]. First-line treatment for metastatic and/or unresectable LMS results in a median progression-free survival of approximately 5 months and an overall survival of 14–16 months [3], significantly affecting patients’ physical and mental health and increasing the overall disease burden. Although immunotherapy has shown promise in various cancers, immune-based treatments for LMS are still under active investigation. So far, only a small subset of patients appears to benefit from these therapies, and the overall efficacy remains unsatisfactory [4–7]. Therefore, identifying biomarkers that reflect immune activity or predict the effectiveness of immunotherapy is essential to guide clinical decision-making, prevent disease progression, uncover new therapeutic targets, and improve treatment outcomes in LMS. The tumor immune microenvironment (TIME) consists of tumor cells, immune cells, and cytokines, which can have either anti-tumor or pro-tumor functions. The interactions among these components shape the dynamics of the anti-tumor immune response [8, 9]. Studies suggest that myeloid-derived cells, particularly monocytes within the TIME, are important indicators of the effectiveness of anti-programmed cell death protein 1 (PD-1) immunotherapy; a higher monocyte ratio is associated with better responses to PD1 inhibitors [10]. Moreover, activated CD103^+^ dendritic cells (DCs) have also been identified as potential biomarkers for anti-PD1 therapy [11, 12]. These findings suggest that monocytes play a critical role in TIME, particularly in mounting an anti-tumor immune response. The complexity of monocyte behavior—including their differentiation and function—is influenced by local factors such as nutrient availability, pH, oxygen levels, and tumor-secreted soluble factors. These environmental cues activate stress-related molecular pathways within monocytes, shaping their phenotype and determining whether they adopt pro-tumor or anti-tumor roles [13]. In essence, the characteristics of the tumor microenvironment define the phenotype of monocytes. Once in tissues, monocytes can differentiate into macrophages or dendritic cells [14]. Blood-borne monocytes display substantial plasticity, with the potential to transition into tumor-associated macrophages [15]. Studying this differentiation process may provide valuable insights into tumor biology and the anti-tumor immune response. However, there remains a significant gap in research exploring the relationship between monocyte differentiation status and patient prognosis or immunotherapy responsiveness in LMS.

Gene mutations—key drivers of abnormal and uncontrolled cellular growth—are hallmarks of cancer [16]. In leiomyosarcoma (LMS), the most commonly mutated genes include *TP53*, *RB1*, and *ATRX* [17]. These mutations can promote or drive tumorigenesis, with individual tumors typically harboring between two and eight such driver mutations [18]. Importantly, gene mutations can also influence immune function and the tumor immune microenvironment (TIME), both of which are closely tied to tumor development. For example, certain mutations may reduce the expression of cell surface antigens, enabling tumor cells to evade immune detection and destruction [19]. Therefore, analyzing the patterns and functional consequences of gene mutations in LMS is crucial for deepening our understanding of disease initiation, identifying novel therapeutic targets, and improving the effectiveness of immunotherapy. In this study, we identified the gene regulatory network underlying monocyte differentiation, as well as key OncoImmune-related hub genes that regulate both monocyte differentiation and LMS progression. Using a machine learning approach, we developed a risk model to predict patient prognosis and immune response in LMS. Additionally, we found that *ATRX* gene mutations significantly impact risk scores, clinical outcomes, and immune function in LMS patients. This discovery highlights *ATRX* as a potential biomarker or therapeutic target, warranting further validation for its role in guiding clinical interventions in LMS.

## Materials and methods

### Data collection

In this study, we analyzed 104 leiomyosarcoma (LMS) samples from The Cancer Genome Atlas (TCGA; GDC, cancer.gov), 87 LMS samples from the GSE159847 [20] dataset available in the Gene Expression Omnibus (GEO; ncbi.nlm.nih.gov/geo), and 142 normal uterus samples from the Genotype-Tissue Expression (GTEx) Project (gtexportal.org). For the TCGA-LMS cohort, we obtained and processed expression matrices (measured as transcripts per kilobase of exon model per million mapped reads, TPM), along with relevant clinical data and mutation profiles, in accordance with the protocols provided by the respective public data repositories. In addition, we obtained single-cell RNA sequencing (scRNA-seq) data related to monocyte differentiation from the GSE218483 dataset in GEO [21].

### Pseudo-time analysis of scRNA seq and identification of differential genes for monocyte differentiation

The “Seurat” package was employed to import and process the scRNA-seq data from GSE218483. Initially, the data underwent quality control to eliminate unqualified cells based on the following criteria: (1) 500 < nFeature_RNA < 4,000; (2) percent < 10%. The samples were then combined using the “harmony” package to address batch effects. Principal component analysis (PCA) was conducted to extract the top 20 principal components (PCs) from the 2000 highest-variance genes. Subsequently, unsupervised clustering was performed using t-distributed stochastic neighbor embedding (t-SNE), allowing for unbiased visualization of cell subpopulations on a two-dimensional map [22]. The FindAllMarkers tool was utilized to identify differential genes between each cluster and all other clusters, applying criteria of |log2 (fold change) | > 0.5 and an adjusted *P* value 0.05. Cell types were annotated using the “SingleR” package [23]. Finally, the “monocle” package was employed to identify distinct states and differential genes during the process of monocyte differentiation [24].

### Identification of the oncoImmune DEGs related to monocyte differentiation and leiomyosarcoma progression

After converting data from FPKM to TPM, differentially expressed genes (DEGs) for 104 LMS patients in the TCGA database and 142 normal uterine samples in the GTEx database were identified using the “limma” package. The intersection of genes associated with monocyte differentiation and LMS progression was then subjected to Gene Ontology (GO) and Kyoto Encyclopedia of Genes and Genomes (KEGG) analyses. Additionally, the STRING database (STRING: functional protein association networks (string-db.org)) was utilized to illustrate the internal connections among the OncoImmune DEGs [25].

### Construction of immune subtypes

The TIMER2.0 database (TIMER2.0 (cistrome.org)) was employed to analyze the composition of TIME in each sample [26]. Following this, unsupervised clustering of LMS samples from the TCGA and GEO databases was performed using nonnegative matrix factorization (NMF). The optimal rank was determined by selecting the first point in the cophenetic coefficient curve that exhibited the steepest decline. Differences in immune cell composition, immune microenvironment, and immune activity among various subtypes were then examined to assess whether distinct subtypes exhibit differing immune functions.

### The establishment and validation of risk model

LMS specimens from the TCGA and GEO databases were utilized to develop a predictive signature, with samples from the GEO database serving as external validation data to assess the model’s reliability. The TCGA samples were divided into a training cohort and a test cohort in a 7:3 ratio. A predictive model was developed using multivariate analysis and LASSO regression, based on the expression matrices of OncoImmune DEGs and patient prognosis in the training cohort [27]. The reliability of the risk prognostic model was further evaluated using decision curve analysis (DCA), a nomogram, and receiver operating characteristic (ROC) curve analysis.

### Evaluation of tumor immune microenvironment

The molecular pathway gene set for correlation enrichment analysis was obtained from GSEA | MSigDB (gsea-msigdb.org). Gene Set Enrichment Analysis (GSEA) was conducted to identify molecular pathways associated with risk scores. Additionally, the relationship between risk scores and TIME was also investigated.

**Figure 1.**
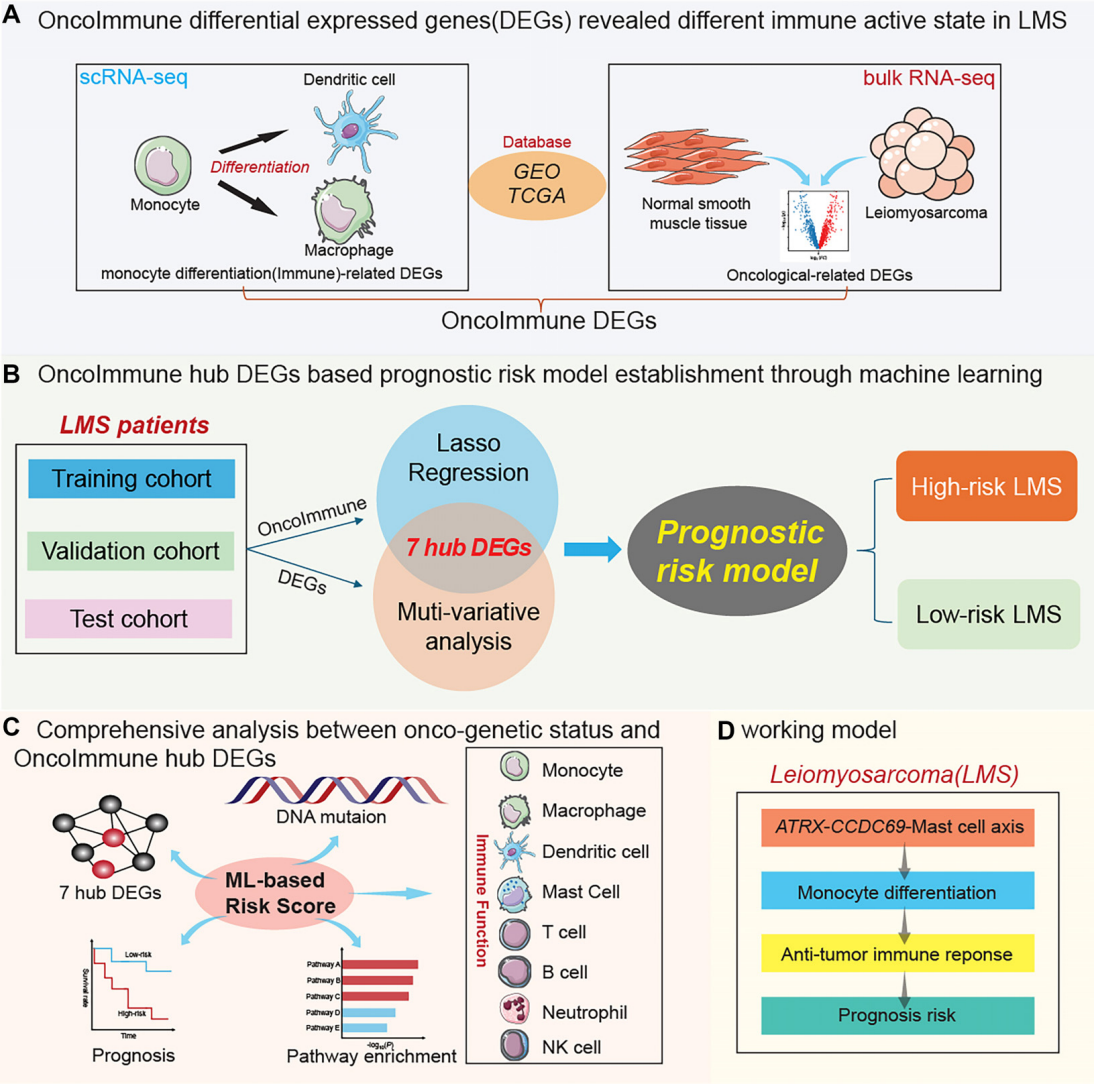
**The whole analysis process of this research.** (A) Integration of scRNA-seq (monocyte differentiation) with bulk RNA-seq from TCGA/GEO to derive OncoImmune DEGs; (B) Construction of an ML-based prognostic model from hub DEGs (LASSO + multivariable analysis) with stratification into high- and low-risk LMS; (C) Linking the risk score to genomic alterations, immune infiltration/composition, and pathway enrichment; (D) Proposed ATRX–CCDC69–mast-cell axis connecting monocyte differentiation to immune activity and prognosis in LMS. LMS: Leiomyosarcoma; DEGs: Differentially expressed genes; scRNA-seq: Single-cell RNA sequencing; TCGA: The Cancer Genome Atlas; GEO: Gene Expression Omnibus; ML: Machine learning.

### Identification of genomic mutation pattern in leiomyosarcoma

Mutation data for LMS were obtained from TCGA and visualized using the “maftools” package [28]. To identify characteristic gene mutation in LMS, we analyzed the relationship between mutation probabilities and risk groups. Subsequently, we examined the differences in risk models, immune function, and prognosis between groups with characteristic gene mutations and those without.

### Relationship between target gene and TIME

To identify the target gene, differential gene expression between mutation and non-mutation groups was analyzed. First, the relationship between the target gene and immune cells within the LMS immune microenvironment was assessed using the “CIBERSORT” package. Subsequently, we explored the association between the target gene and immune function, as well as its involvement in cellular processes related to immunotherapy.

### Statistical analysis

Statistical analysis was performed using R version 4.2.1. Non-parametric tests were applied to compare the two risk categories, with a *P* value of less than 0.05 indicating statistical significance. True associations were determined using Spearman rank correlation analysis.

## Results

### OncoImmune differential expressed genes (DEGs) revealed different immune active state in LMS

**Figure 2.**
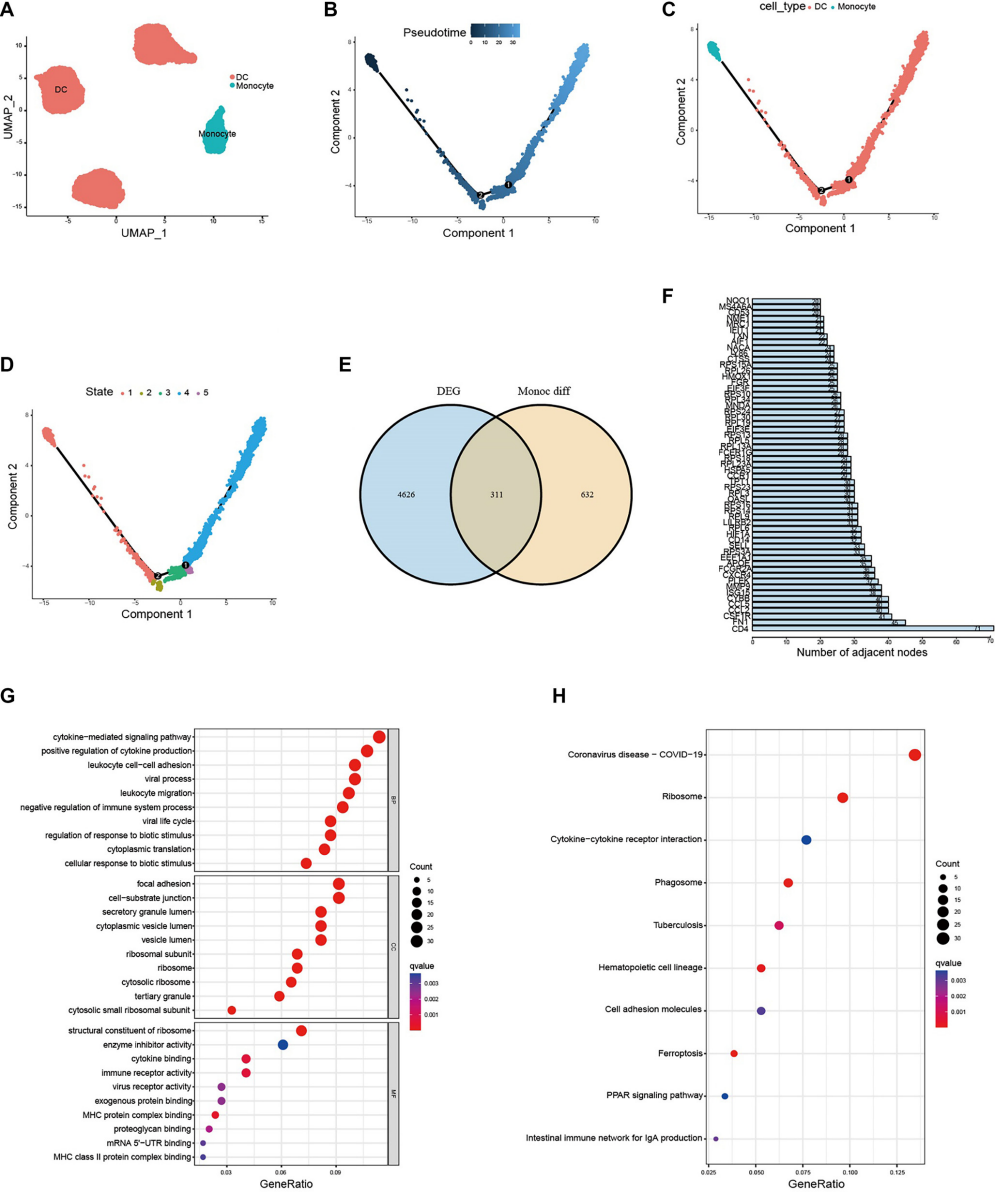
**Identifying theOncoImmune DEGs and exploring their functions.** (A) Identification of 2 types cells during monocyte differentiation; (B–D) Pseudo-time series analysis revealed the different states of monocyte in the process of driving the polarization of differentiation of monocyte; (E) Intersection of genes involved in monocyte differentiation and differentially expressed genes in LMS revealed 311 overlapping genes between the two gene sets; (F) Bar plot showed the genes encoding proteins that had 20 or more nodes interacting with each other (confidence > 0.7); (G) Bubble plot showed the main molecular pathways involved in OncoImmune DEGs based on GO functional enrichment analysis (*P* < 0.05); (H) Bubble plot showed the main molecular pathways involved in OncoImmune DEGs based on KEGG functional enrichment analysis (*P* < 0.05).

Given the critical role of TIME in monocyte differentiation, gene expression levels during this process likely reflect both tumor growth and TIME status. Identifying such gene populations could aid clinical decision-making by serving as biomarkers or potential targets for precision therapy (Fig. 1). Based on data from GSE218483, two cell types—dendritic cells and monocytes—were successfully identified and labeled (Figure 2A). Dendritic cells are believed to derive from monocytes through a specific differentiation process. According to the pseudo-time analysis performed using the “monocle” package, this process was divided into five distinct states (Figure 2B–2D). A total of 943 differentially expressed genes (DEGs) were identified across these states; these genes are hypothesized to play critical roles in monocyte differentiation and to possess immunomodulatory functions (Table S1).

The immune-related DEGs associated with monocyte differentiation were thought to reflect the immune response and tumor survival within the TIME. We hypothesized that similar DEGs may exist in tumor tissue and potentially influence tumor progression, either positively or negatively. To validate this hypothesis, we identified 4937 oncology-related DEGs between normal smooth muscle and LMS samples from the GTEx and TCGA databases (Figure S1). Notably, 311 DEGs overlapped between immune-related and oncology-related DEGs (Figure 2E). These 311 genes were defined as OncoImmune DEGs, which may significantly impact immune responses and tumor progression. Analysis using the STRING database revealed intricate interactions among the OncoImmune DEGs, highlighting their multifunctional roles (Figure S2). Over 60 protein-coding genes exhibited interactions with twenty or more other nodes (Figure 2F). Gene Ontology (GO) and Kyoto Encyclopedia of Genes and Genomes (KEGG) enrichment analyses indicated that these genes are primarily involved in various immune-related pathways, such as cytokine-mediated signaling, cell adhesion molecules, viral processes, and antigen presentation—all of which play essential roles in the LMS TIME (Figure 2G and 2H).

**Figure 3.**
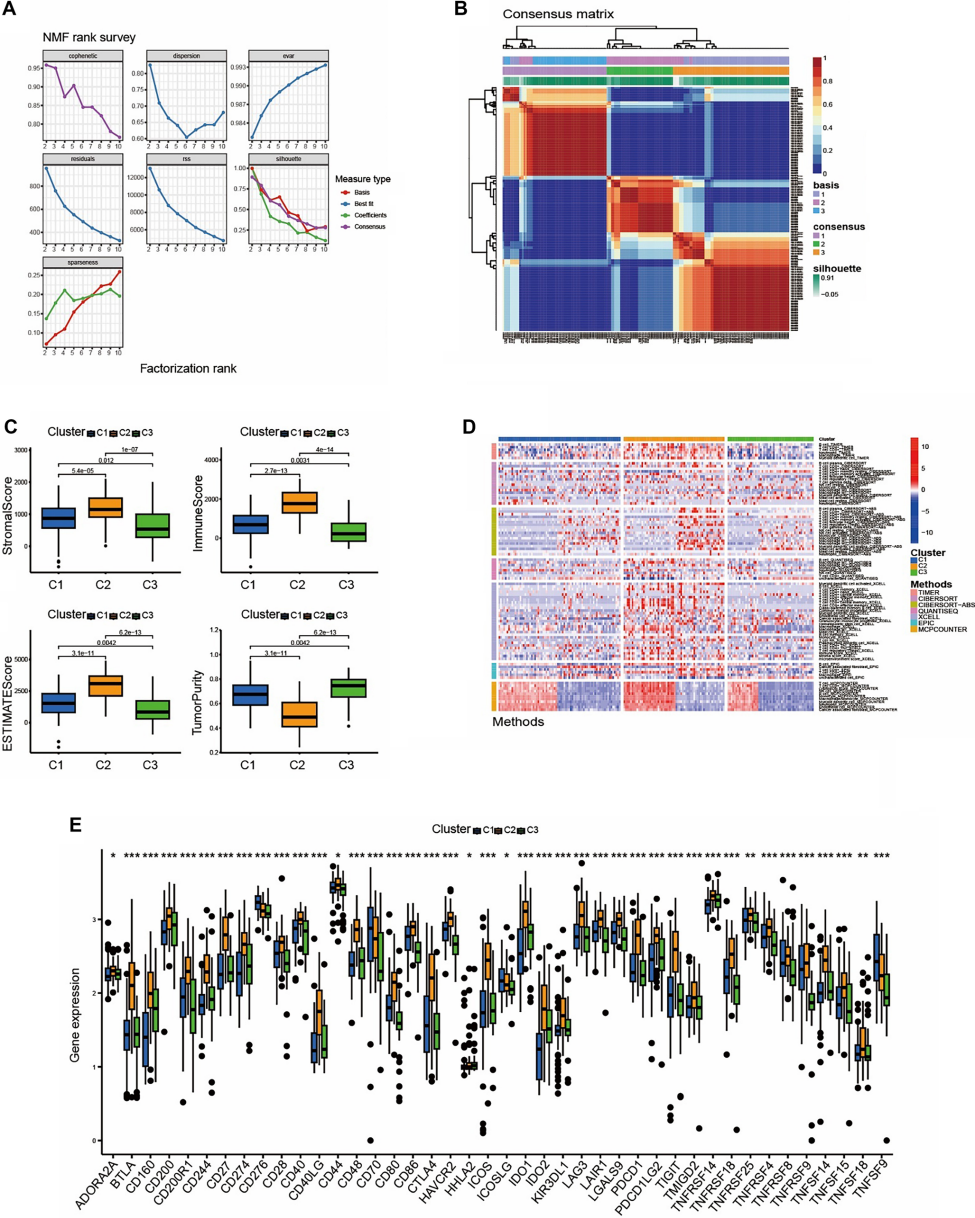
**Construction of immune subtypes.** (A and B) Dimensionality reduction matrix for rank ═ 2 was obtained by applying NMF clustering; (C) Box plot showed the difference in immune microenvironment composition between each immune subtypes, subtype C2 exhibits a more favorable immune microenvironment composition; (D) Heatmap showed immune cell differences between immune subtypes (*P* < 0.05); (E) The bar plot showed the difference in the expression level of immune related genes among immune subtypes, with subtype C2 exhibiting a higher expression levels.

Given that 311 OncoImmune DEGs were identified from the monocyte differentiation process, we anticipated that the differential expression levels of these OncoImmune-related genes would reflect the immune response within the TIME. To investigate this, we classified LMS patients into three subtypes using the NMF algorithm based on the expression profiles of the 311 OncoImmune DEGs (Figure 3A and 3B). Subsequent comparisons revealed significant differences in tumor microenvironment composition among the subtypes. Notably, subtype C2 exhibited a higher stromal score and a relatively lower tumor purity score compared to subtypes C1 and C3 (Figure 3C), indicating a greater presence of stromal components, including immune cells, within the TIME. Additionally, higher ImmuneScore and ESTIMATEScore values—derived from established immune scoring algorithms—confirmed an active immune status in subtype C2 LMS patients (Figure 3C). The heatmap further illustrated that immune cells such as CD4+ T cells, CD8+ T cells, and macrophages were more abundant in the C2 group compared to C1 and C3 (Figure 3D). Furthermore, immune checkpoint genes, including CD274, CTLA4, IDO1, IDO2, and others, exhibited elevated expression levels in subtype C2 relative to C1 and C3, suggesting that this subtype represents an immune-activating or immunologically “hot” phenotype within the TIME (Figure 3E).

### OncoImmune hub DEGs based prognostic model establishment through machine learning approach

**Figure 4.**
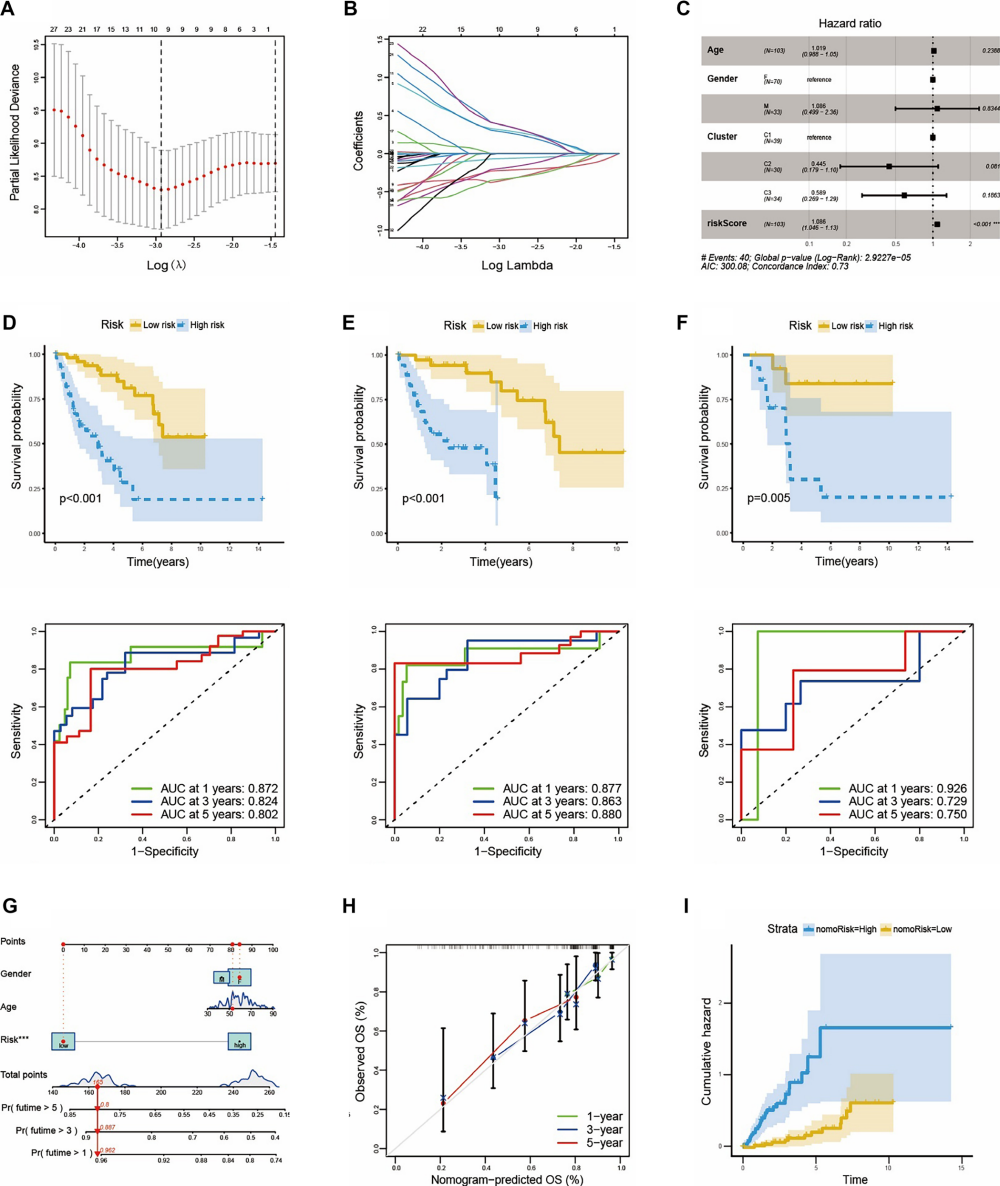
**The establishment of risk model.** (A and B) 7 OncoImmune hub DEGs regulating the differentiation of monocyte and the progression of LMS were screened by LASSO regression and multivariate analysis; (C) Multivariate analysis reveals the relationship between clinical features and risk-score and prognosis; (D–F) The KM curve and time-dependent ROC curve in all TCGA cohort, train cohort and test cohort; (G) Nomogram for 1, 3, and 5-year overall survival of samples combined risk model with clinical features; (H) Calibration curves compare the model prediction probability with the observed probability, the dotted line refers to the ideal nomogram; (I) The cumulative risk curve based on nomogram.

Since the OncoImmune DEGs reflect intratumoral immune activity in LMS patients, it was reasonable to speculate that these DEGs could indicate the risk level of LMS patients, including survival time. To identify essential genes and establish a prognostic model among the 311 OncoImmune DEGs, we first extracted clinical features from LMS datasets. Extra-uterine LMS (excluding uterine LMS) and uterine LMS (uLMS) samples from the TCGA database were used as internal data to create a predictive model, serving as the training cohort and test cohort, respectively. Additionally, LMS samples from the GEO database were selected as external data to validate the model’s reliability. Next, LASSO regression and multivariate analysis were employed to develop a risk predictive model based on the expression matrices of the 311 OncoImmune DEGs and patient prognosis (Figure 4A and 4B). Ultimately, seven hub genes (*CCDC69*, *FLI1*, *RPS23*, *ORAI1*, *CES1*, *APOL6*, *AHNAK*) were selected to construct the prognostic risk model. These genes were derived from overlapping results of two machine learning analyses, and a risk score was generated for each LMS patient. To evaluate the correlation between the risk score and patient prognosis, multivariate analysis was conducted to assess risk factors, including clinical features (age, gender), immune activation state (based on subtype), and the risk score derived from the seven hub genes. As anticipated, the risk score was significantly associated with patient prognosis (Figure 4C), indicating that a higher risk score correlates with poorer outcomes. Although no statistically significant differences were observed in survival across the subtypes based on immune activity, subtype C2 demonstrated better survival than the other two subtypes (*P* ═ 0.081), consistent with earlier findings.

Based on the median risk score in the training cohort, each sample in both the TCGA and test cohorts was classified as either high-risk or low-risk. Kaplan–Meier analysis revealed that patients in the low-risk group exhibited better overall survival than those in the high-risk group across all cohorts. To evaluate the performance of the risk prognostic model, the area under the curve (AUC) of the receiver operating characteristic (ROC) curve was used as a metric. The model showed AUC values greater than 0.8 in all TCGA cohorts, including the training, validation, and test cohorts (Figure 4D–4F). Similarly, the model applied to the external test cohort also demonstrated clinical predictive value for LMS patients, with a significant difference in survival probability between the high-risk and low-risk groups (Figure S3A). However, AUC values in the external test cohort ranged from 0.588 to 0.660 across different survival years, which were relatively lower than those observed in the internal test cohort (Figure S3B). This discrepancy between internal and external datasets may stem from variations in the clinical characteristics of LMS patients.

The clinical features of LMS patients significantly impact prognosis [29], particularly factors such as age, gender, pathology, and metastasis. To address this, we constructed a nomogram model that incorporated risk score, gender, and age to predict one-, three-, and five-year survival in the internal TCGA cohorts (Figure 4G). Notably, the nomogram-predicted overall survival (OS) closely aligned with the observed OS, indicating moderate accuracy in survival prediction (Figure 4H). In addition, the nomogram model enabled calculation of risk scores for each LMS sample. Cumulative risk increased over time for both the nomo-high-risk and nomo-low-risk groups (Figure 4I), with significantly higher cumulative risk observed in the nomo-high-risk group—consistent with the risk score model based on the seven OncoImmune DEGs. For the external validation cohort, the nomogram model incorporated additional clinical features, including grade, differentiation, and metastasis (Figure S3C). This may explain the relatively low AUC values for survival prediction observed when using only the risk score model. Reliability validation and the cumulative risk curve exhibited similar patterns between the internal and external cohorts’ nomogram models (Figure S3D and S3E). In summary, the prognostic model based on a machine learning approach for calculating risk scores can significantly aid in the clinical management of LMS patients. For high-risk LMS patients, intensive or targeted therapies are essential to improve clinical outcomes.

### Molecular and immune characteristics of different risk level groups of LMS based on OncoImmune hub DEGs model

**Figure 5.**
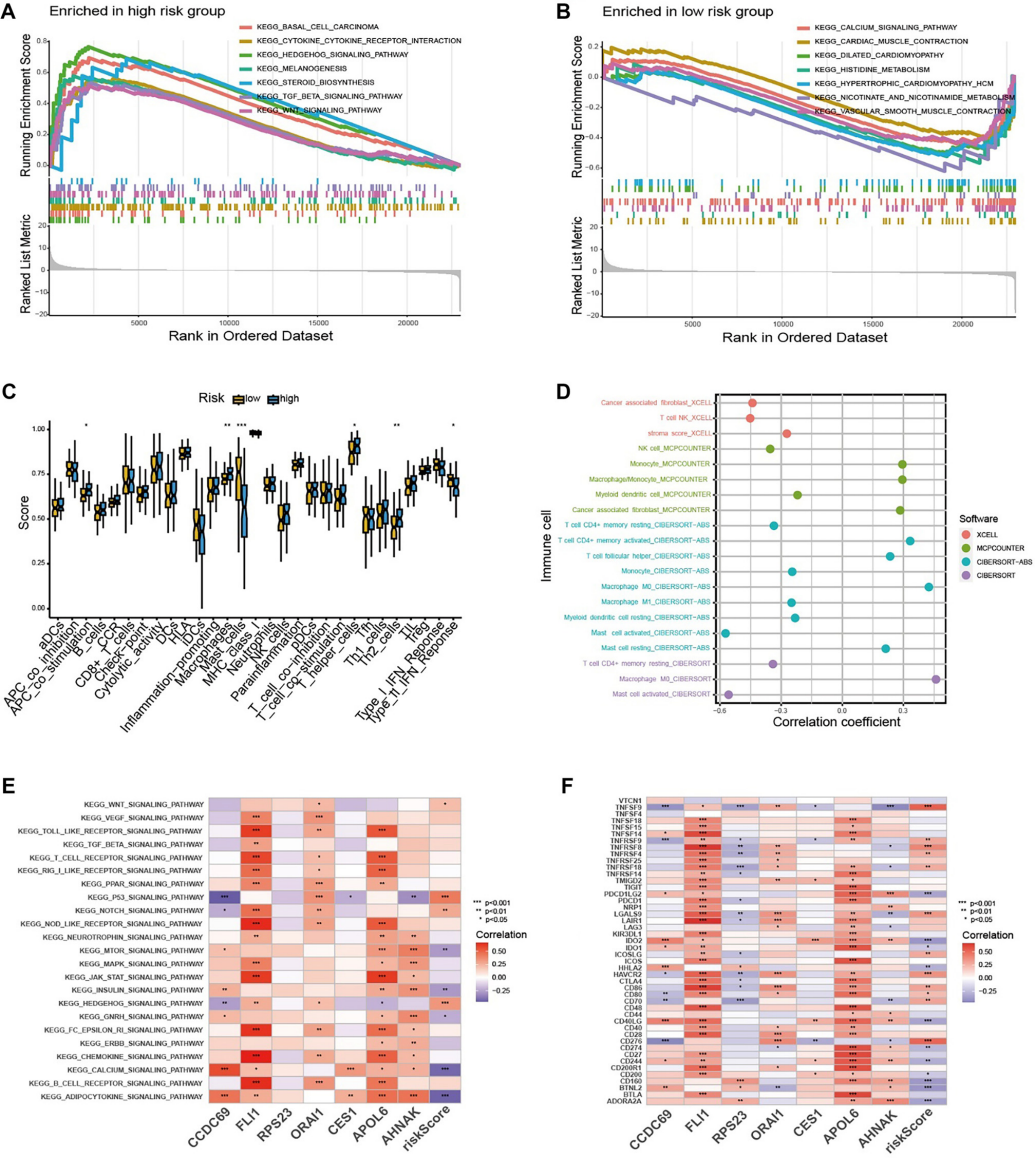
**Correlation analysis between risk-score and TIME.** (A and B) GSEA in high-risk group and the low-risk group; (C) Box plots illustrating immune function scores in low- and high-risk groups. Macrophage function score is elevated in the high-risk group, while mast cell function score is lower compared to the low-risk group; (D) Coefficient plot between immune cells and risk scores; (E) Heatmap of correlation between key signaling pathways and risk scores (*P* < 0.05); (F) The correlation heatmap assessed the relationship between immune checkpoint-related genes and prognostic genes and risk-score.

Although the risk level can be calculated using a machine learning-based prognostic model, the underlying molecular and immune characteristics of different risk groups remain unclear. Gene Set Enrichment Analysis (GSEA) was performed to compare the intrinsic differences between LMS patients across varying risk levels. Signaling pathways—including the transforming growth factor beta (TGF-β) signaling pathway, Hedgehog signaling pathway, and Wnt signaling pathway—were significantly enriched in the high-risk group (Figure 5A). These pathways are essential for cell proliferation, survival, and metastasis in various malignancies, including LMS [30, 31]. Furthermore, pathways such as TGF-β signaling are known to contribute to immune suppression within the tumor microenvironment [32], suggesting that alterations in these pathways may exacerbate disease progression. In contrast, the signaling pathways enriched in the low-risk group included the calcium signaling pathway, cardiac muscle contraction, hypertrophic cardiomyopathy, and vascular smooth muscle contraction—pathways primarily associated with muscle tissue function. This implies that tumor tissue in low-risk patients exhibits fewer abnormalities relative to normal smooth muscle tissue (Figure 5B). Overall, these results highlight the molecular heterogeneity among LMS patients with different risk profiles. Additionally, assessment of immune function scores revealed that the macrophage function score was elevated in the high-risk group, while the mast cell function score was significantly lower compared to the low-risk group (Figure 5C).

To investigate immune characteristics, we employed four well-established algorithms—XCELL, MCPCOUNTER, CIBERSORT-ABS, and CIBERSORT—to generate a coefficient plot illustrating the relationships between immune cell populations and risk scores (Figure 5D). Interestingly, activated mast cells, M1 macrophages, and NK cells were significantly negatively associated with risk scores, while M0 macrophages and resting mast cells exhibited a positive correlation. This suggests that the differentiation of macrophages from M0 to M1 may be linked to a reduced risk score, as M1 macrophages are known to exert anti-tumor effects [33]. These results strongly support the initial hypothesis that monocyte cell differentiation reflects tumor progression. Furthermore, we identified a correlation between risk scores and immune-related signaling pathways, with *FLI1* and *APOL6* showing strong associations with these pathways (Figure 5E). Additionally, risk scores and the seven OncoImmune hub DEGs were significantly correlated with immune checkpoint proteins, particularly *FLI1* and *APOL6* (Figure 5F).

### Relationship between onco-genetic status and OncoImmune hub DEGs model of LMS

**Figure 6.**
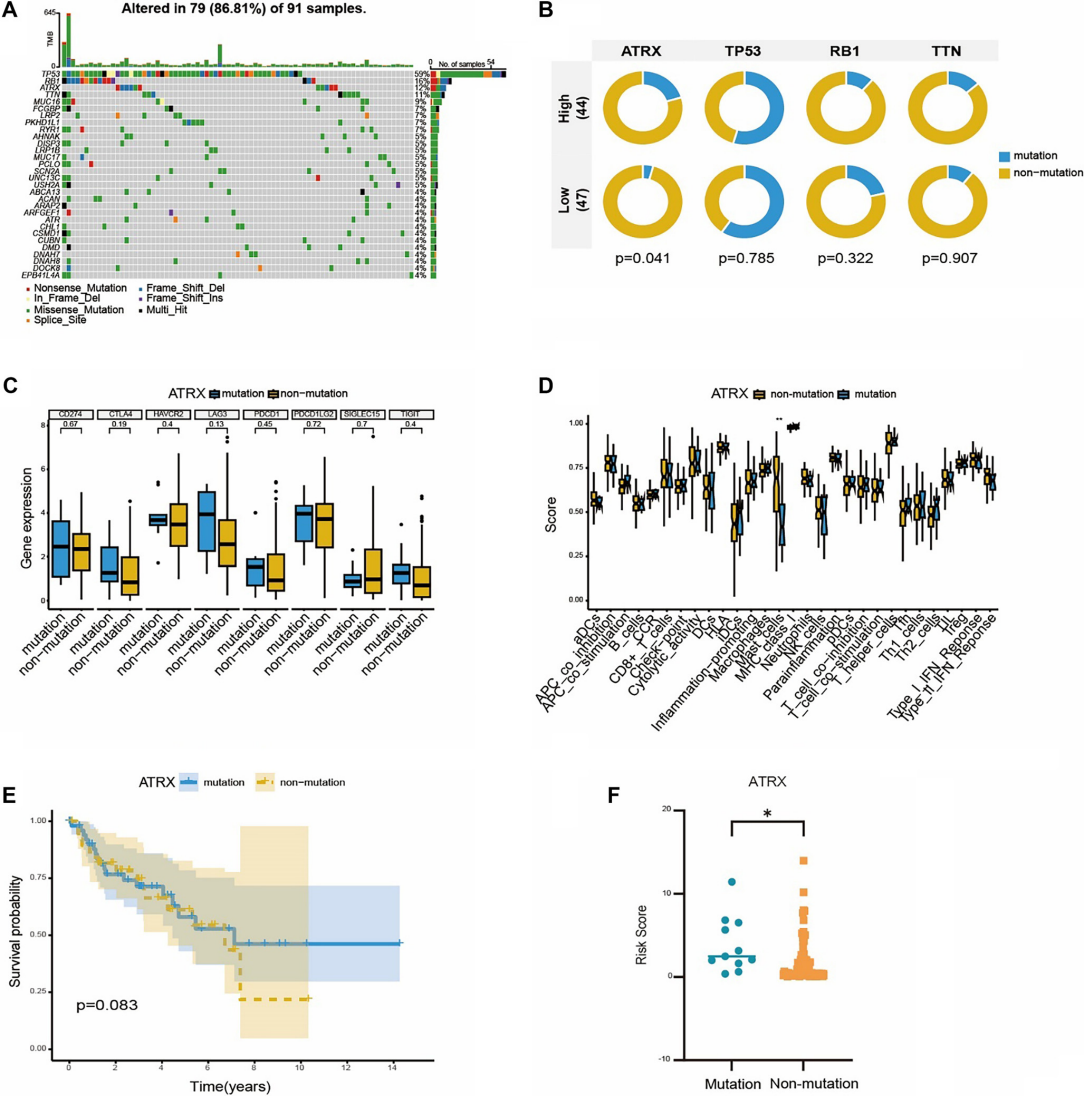
**Identifying the gene mutation and exploring the relationship between gene mutation and TIME as well as risk model in LMS.** (A) Waterfall map of mutation data in LMS samples; (B) Circos plot assessed the relationship between the mutation probability and risk groups, *ATRX* mutations occur more frequently in the high-risk group; (C) Boxplot compared the checkpoint-related genes expression level between *ATRX* mutation and non-mutation groups; (D) Box plot comparing immune function with or without *ATRX* mutation, *ATRX* mutations significantly impair mast cell function; (E) The KM curve in *ATRX* mutation (*n* ═ 11) and non-mutation groups (*n* ═ 80) (*P* ═ 0.083); (F) The difference of risk score between *ATRX* mutation and non-mutation groups, *ATRX* mutations are associated with a higher risk score.

Tumor evolution occurs through the accumulation of mutations in driver genes, including tumor suppressor genes and oncogenes [34]. We hypothesized that mutations in specific genes in LMS could alter risk levels by affecting their normal functions related to immune response modulation, cell proliferation, and survival. Analyzing mutation data from LMS samples in the TCGA, we observed numerous mutations across several genes, with *TP53*, *RB1*, *ATRX*, and *TTN* exhibiting mutation frequencies above 10% (Figure 6A). These mutation frequencies are consistent with previous reports [17, 35]. This analysis not only highlights the unique oncogenic landscape of LMS patients but also reveals shared features with other malignancies. Moreover, these mutated oncogenes and tumor suppressors may significantly influence the risk levels of LMS patients. To evaluate this, we examined the association between oncogenic mutation status and the prognostic risk model based on the OncoImmune hub DEGs. Notably, the mutation frequency of *ATRX* was significantly higher in the high-risk group compared to the low-risk group (Figure 6B), suggesting a potential link between *ATRX* mutation and elevated risk scores. In contrast, other genes such as *TP53*, *RB1*, and *TTN* did not show statistically significant differences in mutation frequency between the two risk groups.

The types of *ATRX* mutations were primarily nonsense, missense, and frameshift deletions, which are classified as loss-of-function mutations (Figure 6A). Inactivating mutations in *ATRX* have been shown to disrupt immune signaling pathways, such as promoting immunosuppressive mechanisms in *IDH1*-mutant gliomas and impairing cGAS-STING signaling in sarcomas [36–38]. We hypothesized that *ATRX* mutations could modulate the expression of genes involved in anti-tumor responses, including immune checkpoint-related genes and components of the tumor TIME. Surprisingly, the expression levels of immune checkpoint-related genes did not differ significantly between the *ATRX* mutant and non-mutant groups (Figure 6C). When evaluating TIME differences using immune function scores, most scores were not statistically significant, with the exception of mast cells (Figure 6D). The immune function score for mast cells was significantly higher in *ATRX* wild-type LMS patients compared to those with ATRX mutations. Mast cells, derived from the myeloid lineage, are closely associated with monocyte differentiation [39]. Their accumulation in and around tumors has been linked to effective immune control, potentially facilitating T cell recruitment [40]. Therefore, we hypothesized that *ATRX* mutations may influence the prognosis of LMS patients—such as risk score (Figure 6F)—through modulation of mast cell activity rather than via immune checkpoint expression or other immune components. However, no significant association was observed between *ATRX* mutation status and overall survival in LMS patients (Figure 6E), which may be attributable to the limited sample size. Thus, further research with larger patient cohorts and experimental validation is needed to confirm this hypothesis.

### ATRX-CCDC69-mast cells axis serving as potential regulatory machinery involving in monocytes differentiation and tumor progression in LMS

**Figure 7.**
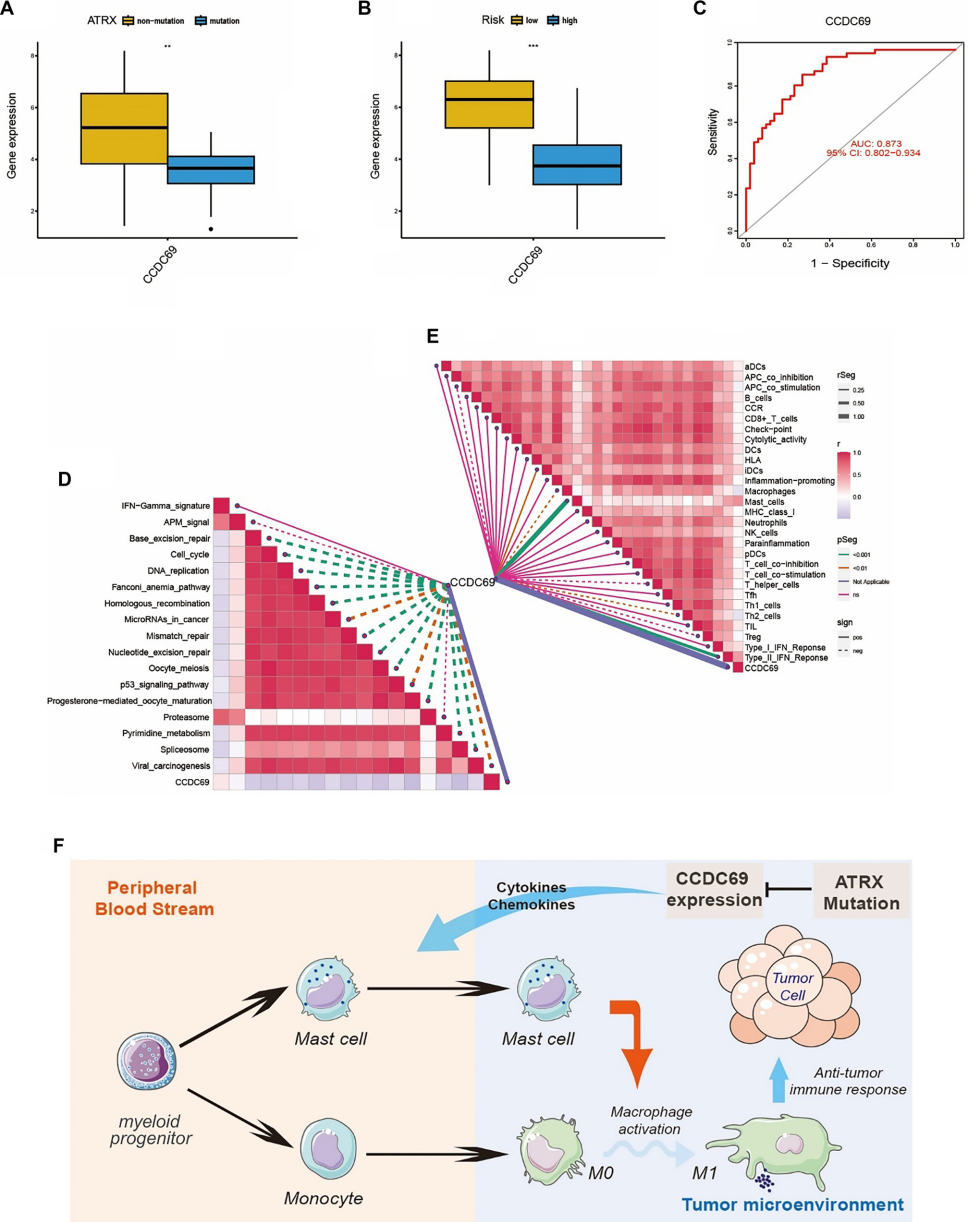
**Correlation analysis of *CCDC69* with prognosis and TIME.** (A) Box plot comparing the expression level of *CCDC69* in *ATRX* mutation and non-mutation groups, the expression level of *CCDC69* is lower in the *ATRX* mutation group; (B) Box plot was used to compare the expression level of *CCDC69* in high-risk and low-risk groups, the expression level of *CCDC69* is lower in the high-risk group; (C) The time-dependent ROC of predicting the risk group of patients based on *CCDC69* expression; (D) Correlations between *CCDC69* and the enrichment scores of immunotherapy-predicted pathways; (E) Relationship between *CCDC69* and immune function in LMS microenvironment, *CCDC69* is positively correlated with mast cell function; (F) A diagram showing the effects and mechanism of *ATRX*-*CCDC69*-mast cell axis in LMS.

As gene mutations represent an initial step in tumorigenesis [41], mutated genes were considered potential regulators of OncoImmune DEG expression, potentially leading to varying risk levels and prognoses. Consequently, we hypothesized that *ATRX* mutations could alter the expression levels of the seven OncoImmune hub DEGs. A comparative analysis was conducted to examine the association between *ATRX* mutation status and the expression level of these hub genes. Interestingly, among the seven genes included in the risk prognostic model, *CCDC69* was the only gene that exhibited a significant difference in expression between the *ATRX* mutant and non-mutant groups (Figure 7A). This finding supports the hypothesis that ATRX mutations may suppress *CCDC69* expression.

Coiled-coil domain-containing protein 69 (*CCDC69*) plays a crucial role in the assembly of the central spindle and the recruitment of midzone components, which are essential for cytoplasmic division in animal cells [42]. *CCDC69* has been identified as a prognostic biomarker in multiple solid tumors [43, 44]. In this study, the expression level of *CCDC69* was significantly higher in the low-risk group compared to the high-risk group (Figure 7B). The AUC from the ROC curve was 0.873 (95% CI: 0.802–0.934) for risk prediction of LMS samples based on *CCDC69* expression (Figure 7C), indicating high accuracy in predicting prognosis using *CCDC69* levels. Changes in *CCDC69* expression resulting from *ATRX* mutation may influence tumor progression and immune responses in LMS tissue. To further support this conclusion, we analyzed the correlation between immune function and *CCDC69* expression, revealing that *CCDC69* exhibited the strongest correlation coefficient with mast cell function within the TIME (Figure 7E). This correlation between *CCDC69* expression and mast cells was consistent with *ATRX* mutation status. Although no direct evidence links *ATRX* mutations to M1/M2 macrophage polarization or function, mast cells are known to interact closely with macrophages in the tumor microenvironment [45]. Collectively, these findings suggest intrinsic differences in the TIME associated with varying *ATRX* statuses and *CCDC69* expression levels, which could inform immunotherapy responsiveness. From a molecular perspective, several predicted immunotherapy-related pathways showed differential correlations with *CCDC69* expression levels (Figure 7D). Notably, the proteasome pathway was among the most significantly enriched pathways associated with *CCDC69* expression (Figure 7D). Interestingly, proteases—among the most abundant proteins in mast cells—play multifaceted roles in their function [46], further supporting a mechanistic link between *CCDC69* expression and mast cell activity.

In summary, *CCDC69* expression is associated with the risk level of LMS patients, reflecting intrinsic differences in TIME, particularly with respect to mast cell activity. The *ATRX–CCDC69*–mast cell axis likely plays a crucial role in modulating the immune response in LMS (Figure 7F), suggesting its potential as a therapeutic target for improving disease management.

## Discussion

Leiomyosarcoma (LMS) is characterized by a high rate of recurrence and distant metastasis [47]. Advanced LMS patients are often treated with first-line chemotherapy, such as gemcitabine or doxorubicin; however, these therapies frequently result in only a limited duration of response [3, 48]. There is an urgent need for novel therapeutic strategies, as current targeted therapies and immunotherapies have not significantly improved long-term prognoses. In this study, we identified 311 OncoImmune differentially expressed genes (DEGs) associated with monocyte differentiation and LMS progression. Monocyte differentiation is influenced by the tumor microenvironment. It has been reported that retinoic acid (RA) produced by sarcoma cells inhibits the expression of *IRF4*, a transcription factor that facilitates dendritic cell differentiation, thereby driving monocytes to differentiate into tumor-associated macrophages (TAMs) [49]. Cho and colleagues [50] demonstrated that cancer-associated fibroblasts (CAFs) activated by cancer cells release IL-6 and GM-CSF cytokines, which synergistically induce monocytes to differentiate into M2 macrophages. These findings are consistent with our results, which show that OncoImmune DEGs are enriched in cytokine- and immune response–related pathways. Differentiated monocytes play a critical role in the tumor immune response, and their differentiation states can reflect the overall immune status of the tumor microenvironment [13]. Our analysis revealed DEGs that are differentially expressed across various monocyte differentiation states. Notably, several of these DEGs appear to be regulated by intrinsic molecular features of tumors during tumorigenesis. Therefore, we integrated immune-related DEGs—genes associated with monocyte differentiation—with oncological DEGs—genes involved in LMS pathogenesis. This approach yielded 311 OncoImmune DEGs, which may offer critical insights into the molecular characteristics of the LMS TIME.

Additionally, we established a machine learning (ML)–based prognostic model that effectively captures the prognosis and immune microenvironment of LMS patients. Machine learning techniques are widely used to construct predictive models from diverse datasets and to identify patterns within large-scale data collections [51]. By integrating data from various omics layers—such as genomics and proteomics—ML algorithms can help elucidate complex biological interactions [52]. In this study, we employed a predictive algorithm based on LASSO regression and multivariate analysis, identifying seven OncoImmune hub DEGs to construct a prognostic model for LMS. The model demonstrated strong internal performance (AUC > 0.80), but more modest performance in external validation (AUC: 0.59–0.66). This decline may be attributed to overfitting and differences in clinical characteristics between the training and external validation cohorts. In a previous study [53], a prognostic model for extremity LMS was developed using machine learning, achieving an external validation c-index of 0.87–0.96. That model incorporated clinical features such as age, race, sex, tumor size, and grade, and was validated on an external cohort of 46 patients. In contrast, our model was constructed based on the expression profiles of OncoImmune DEGs and validated using 87 external cases. Although our model showed only moderate external performance, it offers additional insights into immune-related mechanisms and may support immunotherapeutic stratification of LMS patients beyond conventional prognostic evaluation. The model classified LMS patients into high-risk and low-risk groups. Patients in the low-risk group demonstrated better overall survival and increased activation of immune components, including mast cells, M1 macrophages, and natural killer (NK) cells. These findings suggest that individuals in the low-risk group exhibit a more favorable prognosis and a more active immune response. Furthermore, cytokine-related signaling pathways—such as TGF-β and WNT signaling—were notably enriched in the high-risk group. The TGF-β signaling pathway is particularly important in tumor immune response and progression. Within the tumor microenvironment, TGF-β can inhibit the anti-tumor activity of immune cells [54], promote M2 polarization of tumor-associated macrophages [55], and enhance tumor invasion and metastasis [56]. In conclusion, our ML-based prognostic model not only predicts LMS patient outcomes but also reflects the status of the tumor immune microenvironment. This model may assist in clinical decision-making, particularly regarding immunotherapeutic and targeted treatment strategies in LMS.

Genetic mutation are fundamental driving forces behind tumor development. Numerous studies have shown that LMS frequently harbors mutations in genes such as *TP53*, *ATRX*, *RB1*, and *PTEN* [57, 58], which our findings corroborate. In our study, the most commonly mutated genes in LMS were *TP53*, *RB1*, *ATRX*, and *TTN*. Surprisingly, only the *ATRX* mutation was significantly more frequent in the high-risk group than in the low-risk group, suggesting that it may play a pivotal role in the malignant progression of LMS. *ATRX* (alpha-thalassemia mental retardation X-linked) is known to regulate essential processes such as chromatin remodeling, gene expression, and DNA damage repair, thereby contributing to genomic stability and exerting potent tumor-suppressive functions [59]. Hu et al. [36] found that *ATRX* inactivation led to immune checkpoint upregulation and altered cytokine/chemokine expression, fostering an immunosuppressive response in IDH1^R132H^/TP53^mut^ astrocytoma and enhancing tumor aggressiveness. Similarly, another study reported that reduced *ATRX* expression accelerates tumor growth and promotes immune escape by decreasing the presence of active mast cells in the sarcoma microenvironment [60]. In our study, *ATRX* mutations in LMS were predominantly inactivating. Although they did not significantly impact patient survival (*P* ═ 0.083), these mutations were associated with a higher risk of LMS. Additionally, previous studies have shown that *ATRX* mutations are linked to poor prognosis in uterine LMS (uLMS) patients [61]. We believe that with larger clinical cohorts and future experimental validation, the prognostic relevance of *ATRX* mutations in LMS will become clearer. Apart from downregulating mast cell immune function, this mutation did not appear to significantly affect the expression of immune checkpoint genes or alter the immune activity of other components within the TIME. Notably, a previous study reported that gliomas with *ATRX* mutations are more likely to be infiltrated by immunosuppressive monocytic-lineage cells derived from circulation [62]. Given that our prognostic model partially incorporates monocyte differentiation, it is reasonable to hypothesize that *ATRX* mutations in LMS may induce monocytes to adopt an immunosuppressive state by impairing mast cell function within the TIME, thereby influencing the tumor’s immune response and overall prognosis.

Genetic mutations are often the initial drivers of tumorigenesis [63], and they can significantly influence the expression of OncoImmune differentially expressed genes (DEGs), including those involved in immune responses and tumor progression [64]. In our study, the *ATRX* mutation notably affected the expression of *CCDC69*, one of the seven OncoImmune hub DEGs. Consistent with this, *CCDC69* expression was positively correlated with mast cell immune function. The role of mast cells in tumor progression remains controversial, with studies reporting conflicting findings on their prognostic significance [65]. For instance, in gastric cancer, mast cells promote tumor growth by releasing vascular and lymphatic growth factors [66]. Conversely, in breast cancer, mast cells recruited and activated by tumor cells can induce transcriptional changes in genes such as *SPP1*, *PDCD1*, *IL17A*, *TGFB1*, *KITLG*, and *IFNG*, leading to anti-tumor effects [67]. In our study, both *ATRX* mutations and reduced *CCDC69* expression were associated with higher risk in LMS patients and were linked to diminished mast cell immune function. These findings suggest that mast cells may have a tumor-suppressive role in LMS, aligning with prior reports [60]. Given the known association between *CCDC69* and monocyte differentiation, we hypothesize that mast cells may influence this process in LMS. Supporting this, a previous study [39] showed that mast cells activated by P17—a peptide derived from Tetramorium bicarinatum ant venom—can induce monocyte differentiation into macrophages. Our results further revealed that activated mast cells and M1 macrophages were negatively correlated with high risk scores. This suggests that mast cells may promote monocyte differentiation toward immune-activating phenotypes in LMS, a pathway potentially disrupted by *ATRX* mutations via downregulation of *CCDC69*. Although macrophage polarization and its effects on the tumor microenvironment have been widely studied [68], it remains unclear whether macrophage functional orientation is pre-determined during early monocyte differentiation. Based on our findings, the intrinsic molecular characteristics of LMS—such as specific gene mutation patterns—may play a key role in directing monocyte differentiation toward either pro- or anti-tumor states.

We believe our findings can pave the way for new directions in LMS research. The scientific hypothesis at the core of this study focused on the regulation of immune components and tumorigenesis, enabling a comprehensive analysis of the immune landscape of LMS tumors—particularly in relation to monocyte differentiation. As a proof of concept, our machine learning (ML)-based prognostic risk model, which leverages OncoImmune hub DEGs, demonstrates practical utility in predicting long-term outcomes for individual patients. Beyond the development of the ML-based prognostic tool, we also investigated the underlying mechanisms and characteristics associated with different risk groups in LMS samples. A growing body of research highlights the crucial role of TIME heterogeneity in shaping responses to immunotherapy and influencing clinical outcomes [69, 70]. Additionally, immune cell infiltration and immune-related gene expression significantly affect both the prognosis of LMS and its responsiveness to immunotherapy [61]. In this context, our findings suggest that the *ATRX–CCDC69–*mast cell axis may serve as a relevant immunological and prognostic marker in LMS. This axis offers promising insights for future research aimed at improving anti-tumor immune responses and developing more effective therapeutic strategies.

However, we acknowledge several limitations, including the relatively small sample size, the lack of experimental validation, and the need for further investigation. It is necessary to collect additional samples and conduct experimental research to validate the performance of our prognostic model and further test the ATRX-CCDC69-mast cell axis hypothesis. As more basic and clinical data from LMS patients, particularly those undergoing immunotherapy, become available, this ML-based research on OncoImmune DEGs holds promise for advancing precision medicine and developing more effective targeted immunotherapies.

## Conclusion

This machine learning (ML)-based prognostic risk model utilizing OncoImmune hub DEGs represents promising biomarkers for distinguishing prognosis, molecular characteristics, and immune features in LMS. The *ATRX-CCDC69*-mast cell axis may serve as an immunologically relevant prognostic indicator in LMS patients.

## Supplemental data

Supplemental data are available at the following link: https://www.bjbms.org/ojs/index.php/bjbms/article/view/12342/3913.

## Data Availability

The datasets generated or analyzed during this study are available in The Cancer Genome Atlas (TCGA; https://portal.gdc.cancer.gov/analysis_page?app=Downloads), the Gene Expression Omnibus (GEO; GEO Accession viewer; GEO Accession viewer), and the Genotype-Tissue Expression Project (GTEx; https://www.gtexportal.org/home/downloads/adult-gtex/bulk_tissue_expression).
